# Ultrastructural organization of the thylakoid system during the afternoon relocation of the giant chloroplast in *Selaginella martensii* Spring (Lycopodiophyta)

**DOI:** 10.1007/s00709-023-01888-w

**Published:** 2023-08-23

**Authors:** Andrea Colpo, Sara Demaria, Paola Boldrini, Costanza Baldisserotto, Simonetta Pancaldi, Lorenzo Ferroni

**Affiliations:** 1 Department of Environmental and Prevention Sciences, University of Ferrara, Corso Ercole I d’Este 32, 44121 Ferrara, Italy; 2 Center of Electron Microscopy, University of Ferrara, Via Luigi Borsari 46, 44121 Ferrara, Italy

**Keywords:** Chlorophyll fluorescence, Giant chloroplast, Microphyll, *Selaginella*, Thylakoid, Ultrastructure

## Abstract

Within the ancient vascular plant lineage known as lycophytes, many *Selaginella* species contain only one giant chloroplast in the upper epidermal cells of the leaf. In deep-shade species, such as *S. martensii*, the chloroplast is cup-shaped and the thylakoid system differentiates into an upper lamellar region and a lower granal region (bizonoplast). In this report, we describe the ultrastructural changes occurring in the giant chloroplast hosted in the epidermal cells of *S. martensii* during the daily relocation of the organelle. The process occurs in up to ca. 40% of the microphylls without the plants being exposed to high-light flecks. The relocated chloroplast loses its cup shape: first, it flattens laterally toward the radial cell wall and then assumes a more globular shape. The loss of the conical cell shape, the side-by-side lateral positioning of vacuole and chloroplast, and the extensive rearrangement of the thylakoid system to only granal cooperate in limiting light absorption. While the cup-shaped chloroplast emphasizes the light-harvesting capacity in the morning, the relocated chloroplast is suggested to support the renewal of the thylakoid system during the afternoon, including the recovery of photosystem II (PSII) from photoinhibition. The giant chloroplast repositioning is part of a complex reversible reshaping of the whole epidermal cell.

## Introduction

Lycophytes and euphyllophytes emerged as the earliest divergence in vascular plant phylogeny around 415 million years ago. After the great success of lycophytes in the Carboniferous Period, today lycophytes are a diminutive group, accounting for ca. 1% of extant tracheophytes, with only three families (Lycopodiaceae, Selaginellaceae, Isoëtaceae). In euphyllophytes, a highly conserved feature of leaf micromorphology is the presence of large populations of numerous, small, lens-shaped chloroplasts in the mesophyll cells (Pyke 2009). Likewise, the overall ultrastructure of the thylakoid system in euphyllophytes is almost invariably characterized by two main domains: the appressed thylakoids, forming the granal stacks, and the non-appressed stromal membranes (Solymosi and Keresztes 2012). The complex relations among thylakoid ultrastructure, molecular determinants, and functional consequences are a hot topic in plant cell biology (e.g., Anderson et al. 2012; Pribil et al. 2014; Kirchhoff 2018; Rantala et al. 2020).

Quite interestingly, a much higher variability occurs in lycophytes, particularly in the microphylls of *Selaginella*. Selaginellaceae Willk are a cosmopolitan monogeneric family of lycopods with ca. 750 extant species (Korall and Kenrich 2002; Weststrand and Korall 2016). The general morphology of the extant species has changed little as compared to the fossil records dating back to the Carboniferous Period and is characterized by delicate flattened shoots with dichotomously branching stems and thin microphylls (Korall and Kenrich 2002; Schmidt et al. 2020). In most *Selaginella* species, typically from rainforests, the microphyll is comprised of an upper leaf epidermis formed by large conical cells, a spongy mesophyll, and the flat cells of the lower epidermis (Haberlandt 1888). In species with flat upper epidermis cells, the conical cells form palisade-like tissue under the epidermis (Adame-González et al. 2019; Liu et al. 2022). A comprehensive study by Liu et al. (2020) analyzed the structural variants of the chloroplasts in 76 *Selaginella* species, reporting the occurrence of five categories: monoplastidy in the epidermal conical cells, monoplastidy in the mesophyll conical cells, oligoplastidy, multiplastidy, and vestigial plastidy. The first category is the most frequent and includes cup-shaped, bilobed, or disk-shaped giant chloroplasts. In a subgroup (subgenus *Stachygynandrum*), the cup-shaped chloroplast hosts a unique sub-differentiation of the thylakoid system, with an upper zone formed by parallel lamellae of a few long appressed thylakoids and a lower zone with a typical granal structure. In *S. erythropus*, Sheue et al. (2007) coined the name of “bizonoplast” to describe the organelle. The bizonoplast is considered a special adaptation to a deep-shade environment, where photosynthesis is almost completely assured by the upper leaf cells (Sheue et al. 2007, 2015). In the relative species *S. martensii*, it was shown that the thylakoid architecture of the bizonoplast can be modulated as a long-term response to the growth light regime (Ferroni et al. 2016). The thylakoid organization changes from mostly pseudolamellar under deep shade to mostly granal under high light, but the thylakoid structural zonation defining the bizonoplast is preserved (Ferroni et al. 2016). In general, the thylakoid zonation appears as a very stable feature in the giant chloroplast (Ghaffar et al. 2018). However, quite intriguingly, in *S. martensii* Liu et al. (2020) could not observe any thylakoid dimorphism.

In this paper, we report a morning-to-afternoon change in the microphyll hue of green observed during the autumn–winter in the *S. martensii* plants cultivated at the Botanical Garden of Ferrara (Italy). In the evening, the terminal branches look like a mosaic of dark and light green microphylls. The process occurs reversibly and is due to the relocation of the giant chloroplast inside the epidermal cell. The photomovement of the giant chloroplast is known for some time in *S. martensii*: under weak light, the chloroplast aligns its cup to the cell bottom, but if exposed to intense light, it moves to one of the side cell walls (Zurzycki and Zurzycka 1951; Zurzycki 1952). Light avoidance movements are deemed particularly important in plants adapted to low light as short-term light stress responses (Königer and Bollinger 2012; Howard et al. 2019). In our greenhouse, however, *S. martensii* is grown under the stable shade conditions determined by above-growing plants (irradiance hardly reaches 50 µmol photons m^−2^ s^−1^ at the *S. martensii* level) and, nonetheless, the chloroplast relocates daily. Considering that the bizonoplast is an adaptation for the efficient use of light under shade conditions, we analyzed the changes in chloroplast morphology and, particularly, thylakoid ultrastructure that accompany the organelle relocation, linking them to the variations in thylakoid function, as assessed by in vivo chlorophyll *a* fluorescence emission.

## Materials and methods

### Plant material

From a colony of *Selaginella martensii* Spring (Selaginellaceae) growing in the warm humid greenhouse at the Botanical Garden of the University of Ferrara (44° 50′30′′ N, 11° 37′21′′ E), eight individuals were planted in pots for easier manipulation and analyses. Analyses were performed in autumn–winter, under the normal conditions of the greenhouse. The temperature was of 25–30 °C, the relative humidity was kept stably above 60%. The light environment was natural shade obtained by above-growing plants and followed the natural photoperiod, reaching an incident light (PAR, photosynthetically active radiation) of approximately 40 µmol photons m^−2^ s^−1^ between 12:00 and 14:00 local time. At 17:00, the PAR was under the sensitivity limit of the quantum radiometer. The microphylls were analyzed in the late morning (11:00; dark green leaves), in the early afternoon (14:30; first appearance of light green leaves), and in the late afternoon (17:30; mosaic of light and dark green leaves).

### Macroscopic examinations

To assess the extent of leaf lightning, 5-cm long apical branches were cut and fixed on an envelope made of Bristol board with a rectangular window. The branch was positioned to show the apex and three subsequent nodes in the window. Pictures were taken under artificial ambient light (fluorescent tubes, 5 µmol photons m^−2^ s^−1^) with a Canon Powershot camera. Images were analyzed using the free processing package Fiji (https://imagej.net/software/fiji/). After conversion to 16-bit format, the threshold was adjusted to detect the entire outline of the sample and the plot profile was obtained. The procedure was repeated lowering the threshold to discard the light green portions of the branch, while maintaining the dark green parts. The standardization of picture capturing allowed the use of similar thresholds for all images. The plot profile data were copied to software Origin™ version 2022 (OriginLab, Northampton, MA, USA). The plot profiles were integrated to obtain the whole area and the darker area. By subtraction, the light green area was calculated and expressed as a percentage.

### Microscopic and submicroscopic analyses

For in vivo light microscopy examinations, the microphylls were carefully sampled, mounted in water onto glass slides, and observed with a Zeiss Axiophot microscope under conventional light or epifluorescence (high-pressure mercury vapor lamp); the chloroplasts were visualized by chlorophyll autofluorescence with excitation at 436 nm (band-pass filter BP436/10, low-pass filter LP470). A × 63 planapochromat objective was used.

For transmission electron microscopy, small portions of branches were cut and immediately fixed in a 3% solution of glutaraldehyde prepared in 0.1 M K-Na phosphate buffer, pH 7.2, for 4 h at 4 °C. After washing, the samples were post-fixed with 1% OsO_4_ in the same buffer over night at 4 °C (Ferroni et al. 2016). Samples were subsequently dehydrated with an ascending acetone series, followed by embedding in Durcupan ACM epoxy resin according with routine protocols. Ultrathin Sects. (80 nm) were obtained using a Reichert Ultracut S ultramicrotome and mounted on copper grids. The grids were stained with uranyl acetate and lead citrate. Sections were observed with a Zeiss EM910 transmission electron microscope at the Electron Microscopy Centre, University of Ferrara. Semithin sections (approximately 2-µm thick) were collected on glass slides and stained with 1% toluidine blue for light microscopy examinations. Area of the vacuole was measured with Fiji software in cells sectioned approximately along their longitudinal axis in which the chloroplast was well visible.

The stacking repeat distance (*SRD*) of the upper lamellae and the grana stacks was measured where the thylakoid membranes appeared sectioned perpendicularly to their planes. According to Ünnep et al. (2014), *SRD* was determined as the value of the periodicity of the thylakoids applying Fast Fourier Transformation (FFT) function of Fiji software on the selected area.

### Prompt chlorophyll a fluorescence analysis

The chlorophyll fluorescence induction curves were recorded in the dark-acclimated state using a continuous excitation Handy-PEA fluorimeter (Hansatech Instruments Ltd., UK). Analysis was performed in uncut branches directly in the greenhouse. The leaf-clip was carefully positioned on the branch (measuring area, 4-mm diameter). The steel shutter plate of the clip was then used to allow a 20-min long dark acclimation. Subsequently, a saturating pulse was applied to the sample (0.6 s at 2500 µmol photons m^−2^ s^−1^, peak wavelength of 650 nm). The sensor unit was set to 0.7 gain value for all samples. The OJIP phases were plotted on a logarithmic scale, where O step is considered as *F*_*0*_ at 20 µs (minimum fluorescence, all opened PSII reaction centers), J step is reached at approximately 2 ms, I step at approximately 30 ms, and the P step corresponds to the maximum fluorescence *F*_*M*_. The semiquantitative comparison of the transients, with respect to events reflected in specific phases, was done upon normalizations between the relevant steps. The fluorescence values normalized between O and P are indicated as *V*_*t*_. The fluorescence values normalized between other steps, generically indicated as *X* and *Y*, are indicated as *W*_*X-Y*_. The difference kinetics Δ*W*_*X-Y*_ were plotted to reveal variations in bands hidden between the main steps (Tsimilli-Michael 2020; Bano et al. 2021). Descriptive fluorescence parameters were calculated according to Strasser et al. (2004) and Stirbet and Govindjee (2011). The PSII excitonic connectivity parameters were estimated according to Strasser and Stirbet (2001). The specific energy fluxes per PSII reaction center calculated according to Strasser et al. (2004) and Stirbet and Govindjee (2011) were corrected multiplying each flux by (1 + *C*) to account for the PSII connectivity, where *C* is the curvature constant (Force et al. 2003). The phenomenological energy fluxes per cross section were calculated following Strasser et al. (2004) and Stirbet and Govindjee (2011), assuming that *F*_*0*_ approximates the absorption per cross section. Tsimilli-Michael (2020) pointed out that this assumption may not be safe in all cases, e.g., for the many factors potentially influencing *F*_*0*_. Nonetheless, we are confident that the resulting phenomenological fluxes are reliable, the *F*_*0*_ values being in line with the degree of leaf lightening. All parameters relevant to this report are reported in Table 1 with their definitions.

**Table 1:** Measured and calculated parameters from the fast chlorophyll *a* fluorescence transient

Parameter	Definition
Descriptive parameters of the OJIP transient	
*F*_*0*_	Minimum fluorescence from dark-acclimated microphyll (at 20 µs)
*F*_*M*_	Maximum fluorescence from dark-acclimated microphyll (at plateau)
*F*_*V*_ = *F*_*M*_ − *F*_*0*_	Variable PSII fluorescence
*F*_*V*_/*F*_*0*_	Index of PSII photochemical capacity
*F*_*V*_/*F*_*M*_	Estimate of PSII maximum quantum yield
*V*_*t*_ = (*F*_*t*_ − *F*_*0*_)/(*F*_*M*_* − F*_*0*_)	Relative variable fluorescence at time t (*V*_*K*_ at 300 µs, *V*_*J*_ 2 ms, *V*_*I*_ at 30 ms)
*M*_*O*_ = ∆*V*/∆*t*	Approximate value of the initial slope (trait O-K) of relative variable chlorophyll fluorescence curve *V*_*t*_
∆*V*_*J*_ = 1 − *V*_*J*_	Estimate of the oxidized plastoquinone pool size, also interpreted as the probability with which a PSII trapped electron is transferred from reduced Q_A_ to Q_B_
∆*V*_*I*_ = 1 − *V*_*I*_	Estimate of the relative size of the pool of PSI end electron acceptors, also interpreted as the probability with which a PSII trapped electron is transferred from reduced Q_A_ to PSI final acceptors (ferredoxin, ferredoxin-NADP^+^ oxidoreductase)
∆*V*_*I*_/∆*V*_*J*_	Pool of PSII electron acceptors relative to pool of plastoquinone, also interpreted as the probability with which an electron is transferred from reduced Q_B_ to PSI final acceptors
*Sm*	Normalized area, proportional to the number of electron carriers per electron transport chain
*N*	Turnover number—the number of times Q_A_ becomes reduced and re-oxidized, until *F*_*M*_ is reached
*V*_*K*_/*V*_*J*_	Index of the PSII donor side integrity
Excitonic connectivity among PSII units	
*W* = (*F*_*L*_* − F*_0_)/(*F*_*J*_* − F*_*0*_)	Relative variable fluorescence at the L band, assuming the best proxy for the connectivity calculation at 100 µs
*W*_*E*_ = 1 − [(*F*_*J*_* − F*_*K*_)/(*F*_*J*_* − F*_*0*_)]^2/7^	Model-derived value of relative variable fluorescence at 100 µs in the hypothesis of unconnected PSII units (exponential dependence of fluorescence on time, with *F*_*0*_ fixed at 20 µs)
*C*_*W*_ = (*W*_*E*_ − *W*)/[*W*(1 − *W*_*E*_)]	Curvature constant calculated within the O-J trait
*C* = *C*_*W*_/*V*_*J*_	Curvature constant extended to the whole O-P transient
*p*_*2G*_ = *C*(*F*_*0*_/*F*_*V*_)	Overall grouping probability (all PSII open)
${p}=\frac{{p}_{2{G}}\cdot({F}_{V}/{F}_{0})}{1+{p}_{2G}\cdot({F}_{V}/{F}_{0})}$	Connectivity parameter
*ω* = *p*(*F*_*M*_*/F*_*V*_)	Probability of the connectivity (all PSII closed)
Specific energy fluxes per active PSII reaction center (corrected by curvature *C*)	
$\frac{ {ABS}}{{RC}}=\frac{{M}_{{\varvec{O}}}}{{{V}}_{J}\frac{{F}_{V}}{{F}_{M}}}(1+C)$	Flux of light absorption per PSII (apparent antenna size of active PSII)
*TR*/*RC* = (*M*_*O*_/*V*_*J*_) (1 + *C*)	Maximum trapped exciton flux per PSII
*ET*/*RC* = (*M*_*O*_/*V*_*J*_) ∆*V*_*J*_(1 + *C*)	Electron transport flux from Q_A_ to Q_B_ per PSII
*RE*/*RC* = (*M*_*O*_*/V*_*J*_) ∆*V*_*I*_(1 + *C*)	Electron transport flux from Q_A_ to final PSI acceptors per PSII
*DI*/*RC* = (*M*_*O*_*/V*_*J*_) (*F*_*M*_*/F*_*0*_sg) (1 + *C*)	Flux of energy dissipation per active PSII
Phenomenological energy fluxes per excited cross section	
*RC/CS*_*O*_ = *F*_0_/(*ABS*/*RC*)	Number of active PSII reaction centers per cross section
*TR*/*CS*_*O*_ = (*F*_*V*_/*F*_*M*_) *F*_*0*_	Maximum trapped exciton flux per cross section
*TR/CS*_*O*_ = (*F*_*V*_/*F*_*M*_) ∆*V*_*J*_* F*_*0*_	Electron transport flux from Q_A_ to Q_B_ per cross section
*RE*/*CS*_*O*_ = (*F*_*V*_/*F*_*M*_) ∆*V*_*I*_* F*_*0*_	Electron transport flux from Q_A_ to final PSI acceptors per cross section
*DI*/*CS*_*O*_ = (1 − *F*_*V*_/*F*_*M*_) *F*_*0*_	Flux of energy dissipation per cross section

### Modulated chlorophyll a fluorescence analysis

For the analysis of slow kinetics of chlorophyll fluorescence, a Junior-PAM (pulse amplitude modulation) fluorometer was used (Walz, Effeltrich, Germany). The branches were selected under low ambient light in the laboratory and positioned under the fibreoptics probe. Subsequently, the room light was switched off and the only light source was a very dim green safe light. After 30-min dark acclimation, the measuring light was switched on and the fluorescence emission allowed to stabilize, then *F*_*0*_ and *F*_*M*_ were determined (saturation pulse of 0.6 s). Subsequently, an induction protocol was applied exposing the microphylls to 820 µmol photons m^−2^ s^−1^ for 285 s; every 15 s, the chlorophyll fluorescence yield *F* was recorded, and a saturation pulse was applied to determine the *F*_*M*_*’* maximum fluorescence in the light-acclimated state. The fluorescence values were combined to calculate three complementary quantum yields according to Hendrickson et al. (2004): actual PSII photochemistry *Y(PSII)* = (*F*_*M*_*’ − F*)/*F*_*M*_*’*; non-regulatory dissipation *Y*(*NO*) = *F/F*_*M*_; regulatory thermal dissipation *Y*(*NPQ*) = *1 − Y*(*PSII*)* − Y*(*NO*). Moreover, Stern–Volmer *NPQ* = (*F*_*M*_* − F*_*M*_*’*)/*F*_*M*_*’* was calculated.

### Data treatment

The statistical treatment of data was done with Origin™ version 2022. Analysis of variance with significance threshold fixed at 0.05 was used to compare multiple samples, followed by post-hoc Tukey’s test. The same software was used for the analytical treatment of the fluorescence traces and for graphing.

## Results

### Progressive lightening of microphylls

**Fig. 1:**
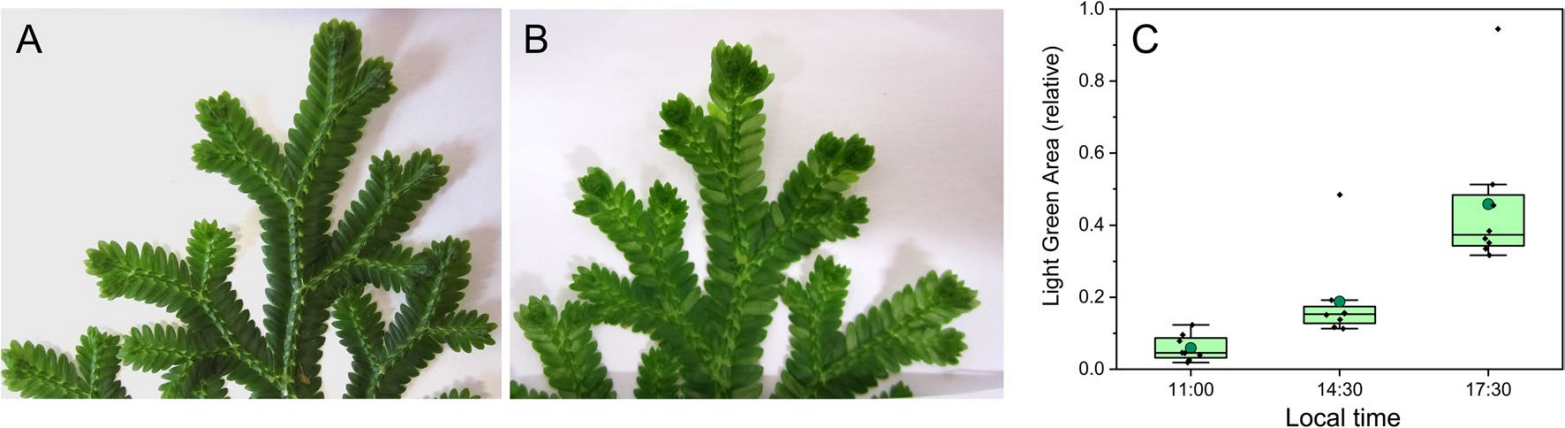
Aspect of microphylls in terminal branches of *Selaginella martensii*. **A** A representative sample collected in the morning shows uniformly dark green microphylls. **B** A representative sample collected in the late afternoon exemplifies the occurrence of many light green microphylls. **C** Quantification of the light green area. In the box charts, the box size is limited by the 25th and 75th percentiles, with whiskers at the 5th and 95th percentiles. The green circle inside the box is the mean, the horizontal segment is the median; all data points are shown as obtained from 8 independent plants

As typical for lycophytes, *S. martensii* shoots are characterized by a dichotomous branching, with one of the two branches being dominant. The species is anisophyllous, with two rows of minute dorsal microphylls and two rows of larger ovate ventral microphylls in an opposite arrangement, which minimizes the mutual leaf shading (Fig. 1A, Dengler 1983). In the late morning (11:00), the colour of *S. martensii* microphylls was dark green and quite uniform along the branches (Fig. 1A). Starting from the early afternoon, some changes in green hues started to become evident, with a progressive lightening. At 17:30, many microphylls were light green, although they were not uniformly distributed along the branches (Fig. 1B). There was no obvious regularity in the distribution of the light green microphylls. The average extent of the light green areas passed from 6% in the morning to 45% in the late afternoon (Fig. 1C). The phenomenon occurred every day and was observed in all the analyzed plants.

### Morning-to-afternoon relocation of the giant chloroplast

**Fig. 2:**
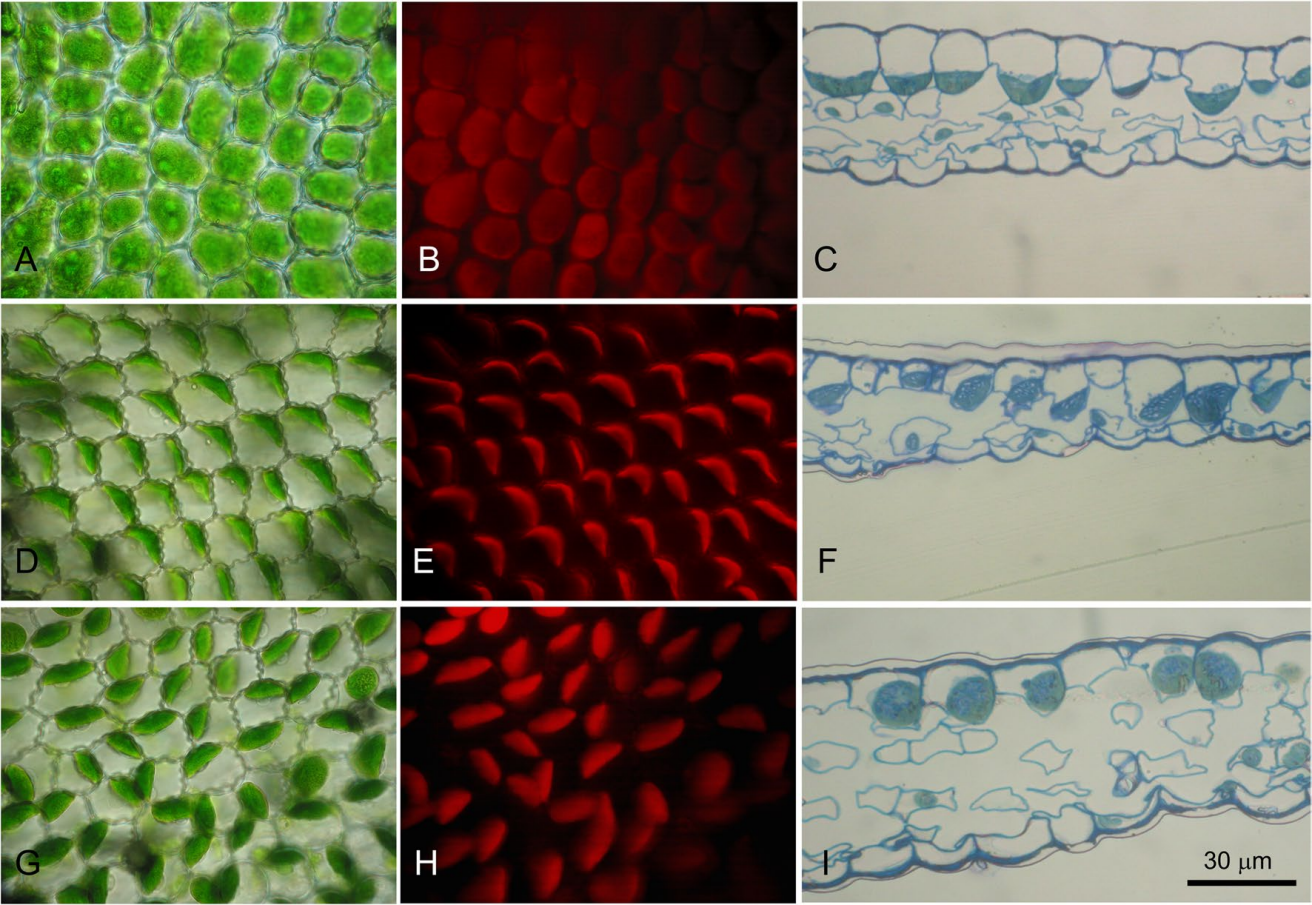
Micrographs of microphylls of *Selaginella martensii*. In the morning, a top view of the dark green microphylls under transmitted white light shows the coherent layer formed by the bright green upper epidermal cells (**A**); each cell hosts a giant chloroplast emitting red chlorophyll fluorescence (**B**); in cross section, the upper page of the microphyll is formed by one layer of conical cells, each containing one giant chloroplast laying at the cell bottom; underneath, the microphyll shows a mesophyll of irregularly shaped mesophyll cells and a monolayered lower epidermis (**C**). In the early afternoon, the light green microphyll areas show flatter chloroplasts relocated towards one radial cell wall with the same orientation in all cells (**D**, **F**) and exhibiting a bright red fluorescence (**E**). In the late afternoon, the light green areas present a random orientation of the disk-shaped chloroplasts, which preserve their fluorescence (**G**, **H**); in cross section, the upper epidermal cells have lost their conical shape and each of them contains one giant globular chloroplast and a visibly smaller vacuole (**I**)

The ventral microphylls were observed in vivo under a light microscope to understand the cause for the colour change. In the morning, the upper epidermal cells appeared as previously described in *S. martensii* and other *Selaginella* species characterized by monoplastidy (Sheue et al. 2007; Ferroni et al. 2016; Liu et al. 2020). In particular, the top view of the microphyll showed uniformly green epidermal cells because of the giant chloroplast, as also evident from the red chlorophyll fluorescence (Fig. 2A–B). In transverse section of fixed samples, the microphyll comprised three cell layers, i.e., the upper epidermis, a mesophyll with sparse lobed cells, and the lower epidermis (Fig. 2C; Ferroni et al. 2016). In the conical upper epidermal cells, one cup-shaped giant chloroplast laid at the cell base, while most of the cell volume was occupied by a large vacuole. The other cell layers contained small lens-shaped chloroplasts. In the early afternoon (14:30), the upper epidermal chloroplast in the light green zones had changed its position inside the cell. Now it laid almost flattened against one radial cell wall, keeping the same orientation in all neighboring cells (Fig. 2D). The chlorophyll fluorescence was particularly bright (Fig. 2E). The new, ordered chloroplast positioning also emerged in the leaf cross sections, which also showed a tendency to a less defined conical shape of the epidermal cells (Fig. 2F). Despite the most extensive light green areas, microscopy observations made in the late afternoon (17:30) showed a less ordered organization of the chloroplasts in neighboring epidermal cells. They were still shifted to one side of the cell but were less flat, and their orientation in adjacent cells was variable (Fig. 2G). The intensity of their fluorescence was intermediate between the chloroplasts in the dark green regions and the light green regions observed in the early afternoon (Fig. 2H). In cross section, inside each cell, the single giant chloroplast was from globular to discoidal in shape (Fig. 2I). In the afternoon samples, the upper epidermal cells appeared much more irregular in shape than in the morning, i.e., not clearly funnel-shaped (Fig. 2F, I). The area occupied by the vacuole in cross sections revealed a reduction by 26% from the morning to the afternoon, without differences between early and late afternoon samples: 245 ± 44 µm^2^ in the morning, 182 ± 42 µm^2^ in the early afternoon, and 181 ± 44 µm^2^ in the late afternoon (*N* = 21–29 cells).

### Ultrastructure of the giant chloroplast

**Fig. 3:**
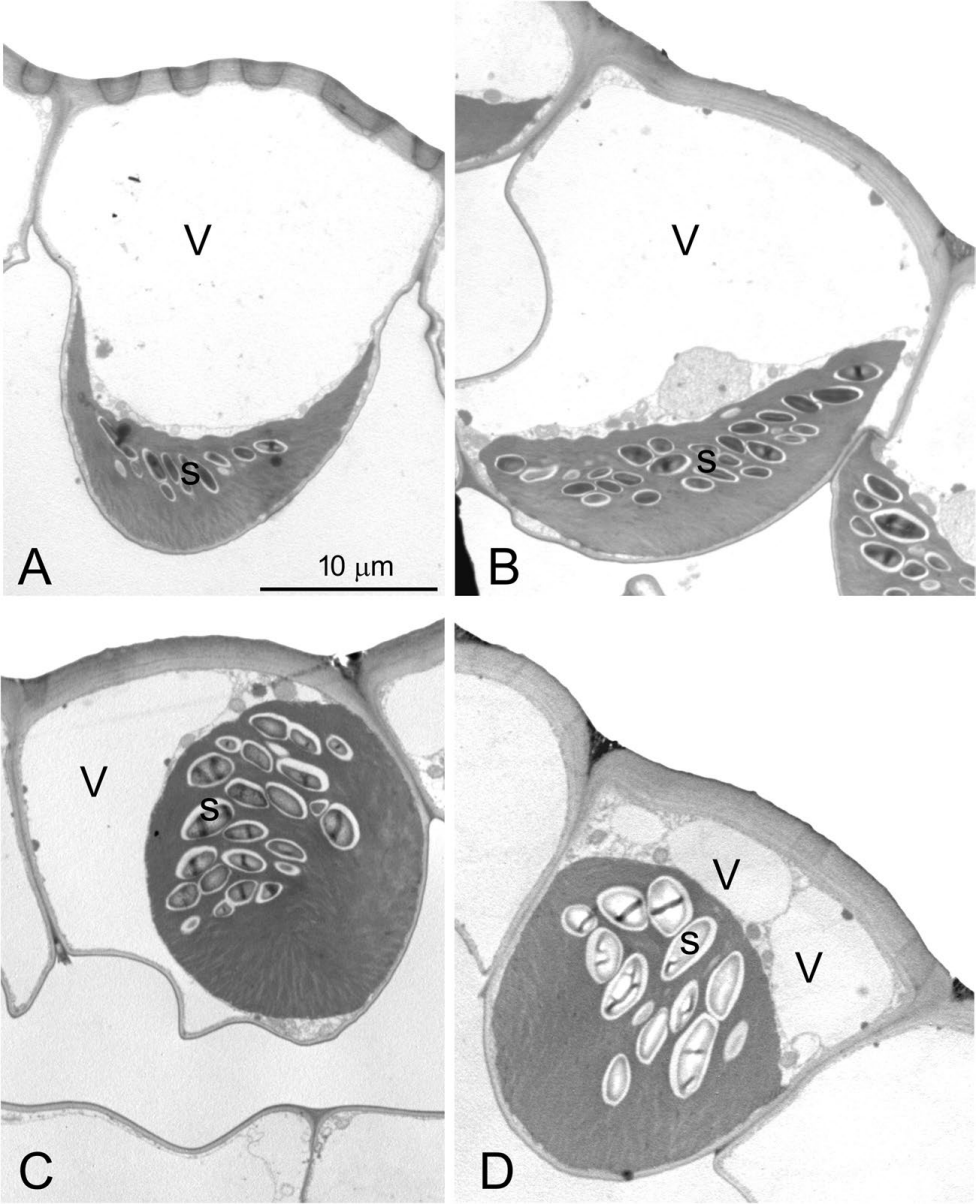
Ultrastructure of the giant chloroplast in the upper epidermal cells of *Selaginella martensii* microphylls. **A** In the morning, an epidermal cell has a typical funnel shape and the cup-shaped chloroplast lays at the cell bottom, with its upper concavity facing the large vacuole; the layer of starch granules is visible. **B** A chloroplast in a light green area in the early afternoon is located towards one radial cell wall opposite to the vacuole; the organelle is particularly rich in starch granules. The cell is more irregular in shape. **C** In the late afternoon, a cell shows an irregular lower portion, in which the cell wall is partly folded. A globular chloroplast with many starch grains faces laterally the vacuole, having lost its upper concavity. **D** Compared to **C**, a perpendicular section of the chloroplast reveals the longitudinal structural homogeneity of the organelle structure. The more peripherical section of the cell is also evidenced by the cytoplasm with organelles above the chloroplast. *s* starch granules, *V* vacuole

In the morning, the chloroplast was surrounded by a thin cytoplasm layer and laid in the lower part of the cell with its concavity facing the vacuole (Fig. 3A). It contained several small starch granules distributed in a layer roughly following the upper outline of the organelle. As already evident from the light microscopy, in the early afternoon, the epidermal cell started to lose its conical shape, and the chloroplast was repositioned flattened towards one radial cell wall; starch granules were more abundant and still forming a layer (Fig. 3B). In the late afternoon, the cell was no longer conical, and its lower part appeared quite irregular because of the partly folded cell wall. The chloroplast became globular in shape. In some sections, the lateral positioning of the organelle, facing the vacuole on the side opposite to the cell wall, was clearly evident (Fig. 3C); given the apparently random lateral positioning of the organelle in neighboring cells, in some cases, the chloroplast appeared at the cell bottom (Fig. 3D). As compared to the morning and early afternoon chloroplasts, the starch granules were larger and not distributed in a well-defined layer (Fig. 3C-D).

**Fig. 4:**
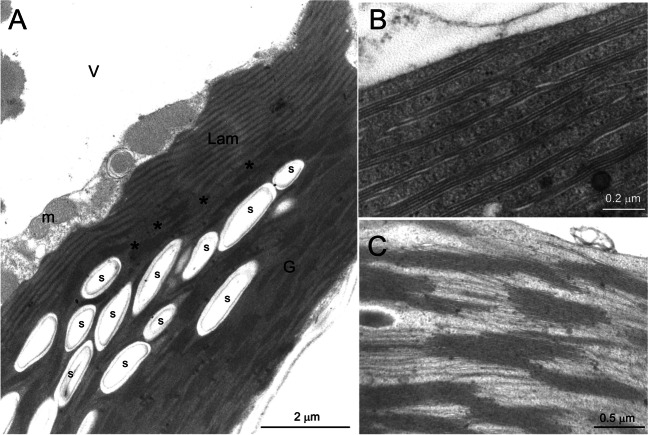
Morning ultrastructure of the thylakoid system in the giant chloroplast in the upper epidermal cells of *Selaginella martensii* microphylls. **A** Overall aspect of the thylakoid system, showing the unique organization of the bizonoplast, with an upper lamellar region and a lower granal region. A layer of starch granules roughly divides the two regions and just above it a granal-to-lamellar transition region is visible (asterisks). **B** Detail of the lamellar region, showing the ordered organization of parallel thylakoid lamellae, each formed by 3–5 appressed thylakoids. **C** Detail of the grana region. *G* grana region, *Lam* lamellar region, *m* mitochondria, *s* starch granules, *V* vacuole

In the morning samples, the observation of the giant chloroplast at higher magnification showed the sub-differentiation of the thylakoid system in two regions (Fig. 4A). In the upper zone, several long straight lamellae formed by 3–5 appressed thylakoids crossed the stroma in a well-ordered parallel arrangement, devoid of grana stacks (Fig. 4B); the thylakoid lumen was well visible especially in the delimiting thylakoids of the lamella. In the lower zone, the thylakoid organization was granal, with large stacks connected by single stroma thylakoids (Fig. 4C). The lumen was visible in the stroma-exposed thylakoids but very narrow in the grana cores. The grana were only seldom observed as individual stacks but tended instead to be in continuity with each other (Fig. 4C). Just above the layer of starch granules, a transition region was visible, in which the lamellar organization gradually became granal (Fig. 4A). The dimorphic thylakoid ultrastructure defines the giant chloroplast of *S. martensii* as a cup-shaped bizonoplast (Sheue et al. 2007; Ferroni et al. 2016).

**Fig. 5:**
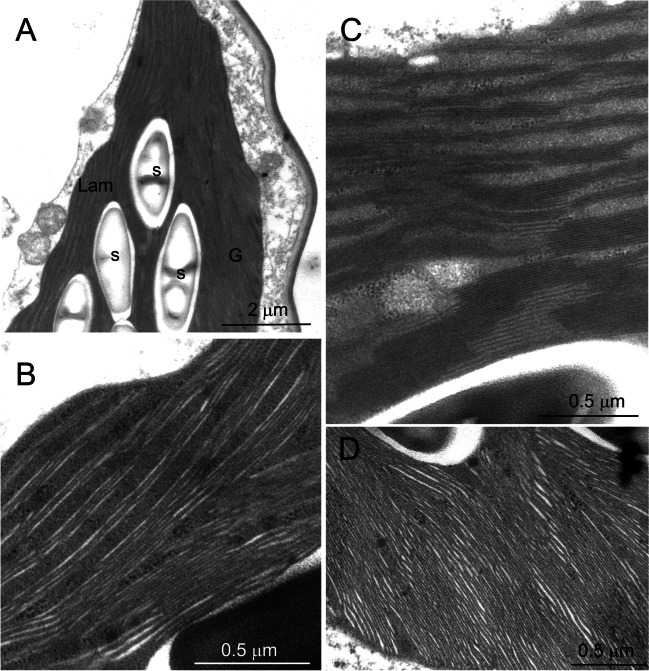
Early afternoon ultrastructure of the thylakoid system in the giant chloroplast of the upper epidermal cells of *Selaginella martensii* microphylls turned to light green. **A** Overall aspect of the thylakoid system, in which the thylakoid zonation is still visible at the two sides of the starch granule layer. **B** Detail of the lamellar region; note the very dense stroma. The thylakoid lumina appear as white regions within the thylakoid lamellae. **C** Detail of a granal-to-lamellar transition zone, extending up to the chloroplast envelope; a clear zonation is however not recognizable. **D** Detail of the granal region with a very packed thylakoid system. *G* grana region, *Lam* lamellar region, *s* starch granules

In the early afternoon, the chloroplast positioned against one radial side wall and the thylakoid system appeared very compact and immersed in a dense stroma (Fig. 5A). Nevertheless, the thylakoid zonation was overall preserved. Beside normally structured lamellar zone, less ordered portions were observed, with an intermediate organization between long lamellae and grana, extending all the way under the plastid envelope (Fig. 5B–C). In the granal region, the thylakoid stacks were close to each other and hardly distinguished as individual units (Fig. 5D).

**Fig. 6:**
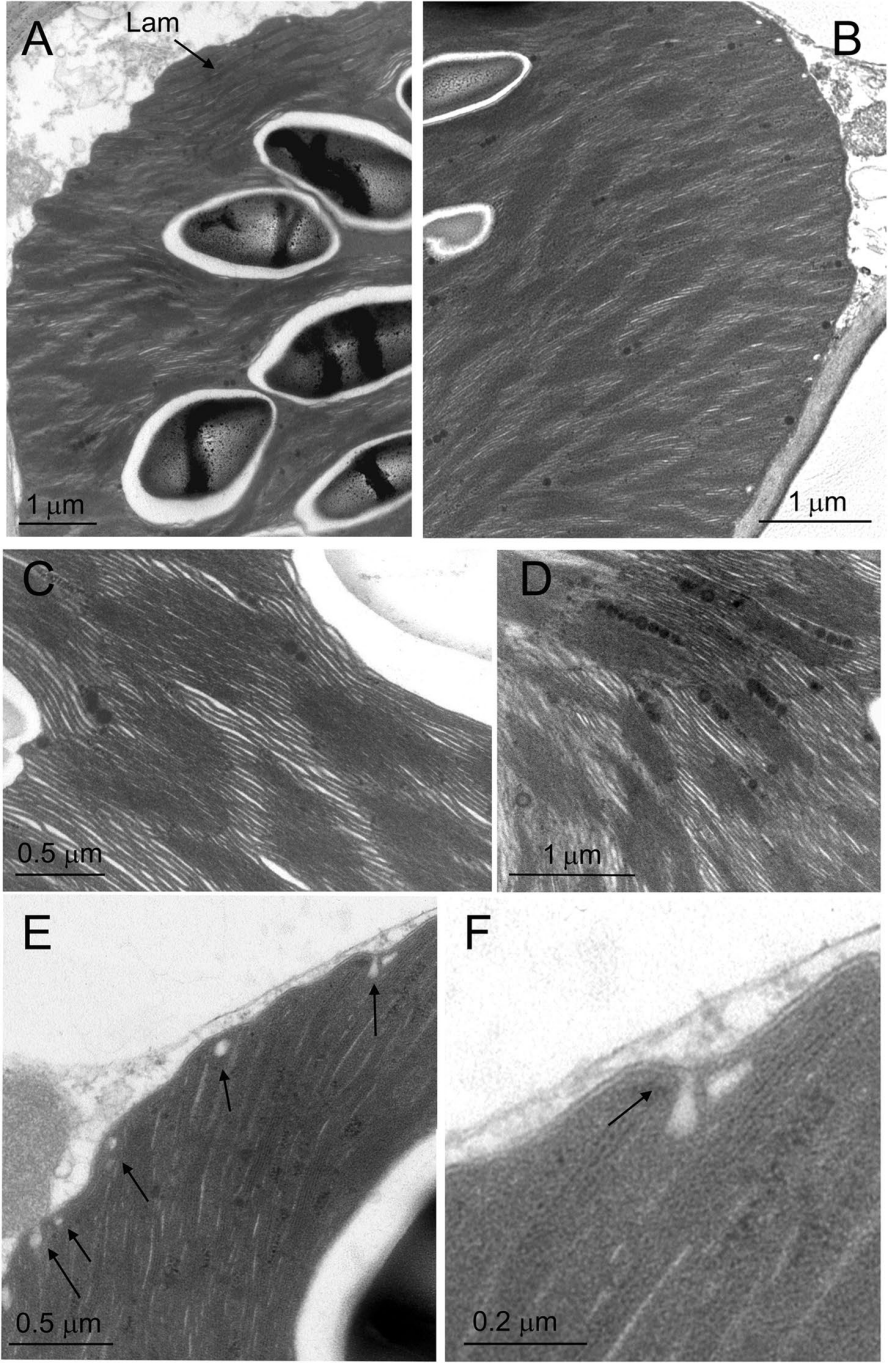
Late afternoon ultrastructure of the thylakoid system in the giant chloroplast in the upper epidermal cells of *Selaginella martensii* microphylls turned to light green. **A** Overall aspect of the thylakoid system, in which only remnants of the lamellar zone are visible. **B** A view of the granal organization. **C** Detail of grana, which are very close to each other and in continuity. The thylakoid lumina are poorly visible both in the grana stacks and stroma lamellae. **D** Detail of the thylakoid system showing the abundance of osmiophilic plastoglobuli, here mostly organized in rows. **E** A micrograph taken at the chloroplast periphery. Note the very low contrast between thylakoids and dense stroma; several vesicles are localized under the plastid envelope (arrows). **F** In a high magnification detail, a vesicle is budding from the inner membrane of the envelope, to which it is still connected by an isthmus (arrow). The thylakoid membranes appear stuck leaving no space for the lumen. *Lam* lamellar region

**Fig. 7:**
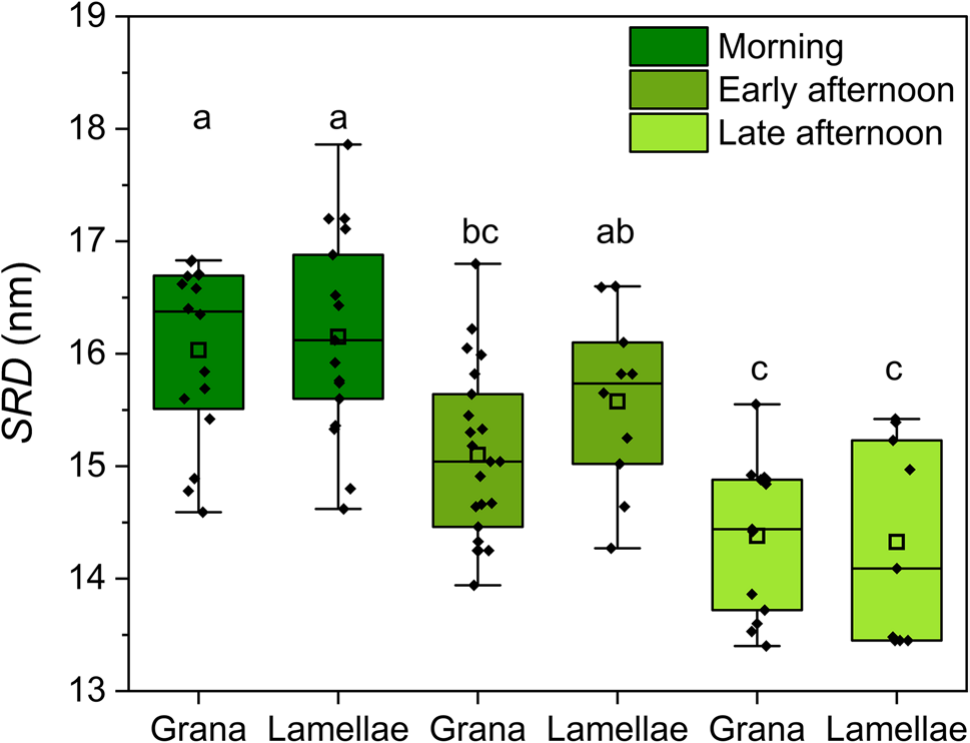
Changes in the thylakoid stacking repeat distance (*SRD*) in the giant chloroplast of *Selaginella martensii* from the morning to early and late afternoon. Analysis was performed on grana stacks and on the lamellar region. The determinations are indicated as black diamonds, the box size is determined by the 25th and 75th percentiles with whiskers at the 5th and 95th percentiles. The square inside the box is the mean, the segment is the median. Means that do not share a letter are statistically different at *P* < 0.05 according to Tukey’s test

In the late afternoon, the chloroplasts had almost completely lost the thylakoid zonation. Only some remnants of the lamellar region could be sometimes observed in limited portions at the top part of the organelle (Fig. 6A). The thylakoid system was formed by a network of grana stacks, not only connected by stroma thylakoids, but also in continuity with each other (Fig. 6B). The stroma thylakoids were also laying very close to each other, giving rise to a packed thylakoid architecture (Fig. 6C). The thylakoid lumen was extremely narrow. The shrinkage of the thylakoid lumen from the morning to the late afternoon was quantified measuring the *SRD*, which is defined as the average thickness of the thylakoid with the neighboring partition gap (Mazur et al. 2021). *SRD* decreased from ca. 16 to 14.3 nm, without significant difference between thylakoids appressed in grana or in long lamellae, also when the latter were reduced to remnants (Fig. 7). Overall, the organelle could be classified as a giant disk-shaped chloroplast without thylakoid ultrastructural dimorphism (Liu et al. 2020). Osmiophilic plastoglobuli, sometimes organized in rows, were associated with the thylakoids (Fig. 6D). In the most peripheral region of the chloroplast, the thylakoid outline was hardly recognized because the very appressed thylakoids, even stuck at their luminal side, did not contrast sufficiently on the surrounding dense stroma (Fig. 6E–F). Interestingly, many small clear vesicles were observed underneath the plastid envelope, budding from the inner membrane (Fig. 6E–F). The vesicles were not perfectly round in shape and appeared as solitary units with a diameter of ca. 50 nm.

### Thylakoid membrane function

We hypothesized that the marked daily changes in the overall thylakoid architecture had a measurable impact on the photosynthetic membrane function; in particular, a very high thylakoid packing could impact on light harvesting and electron transport. We probed the system using two complementary methods based on chlorophyll *a* fluorescence emission.

**Fig. 8:**
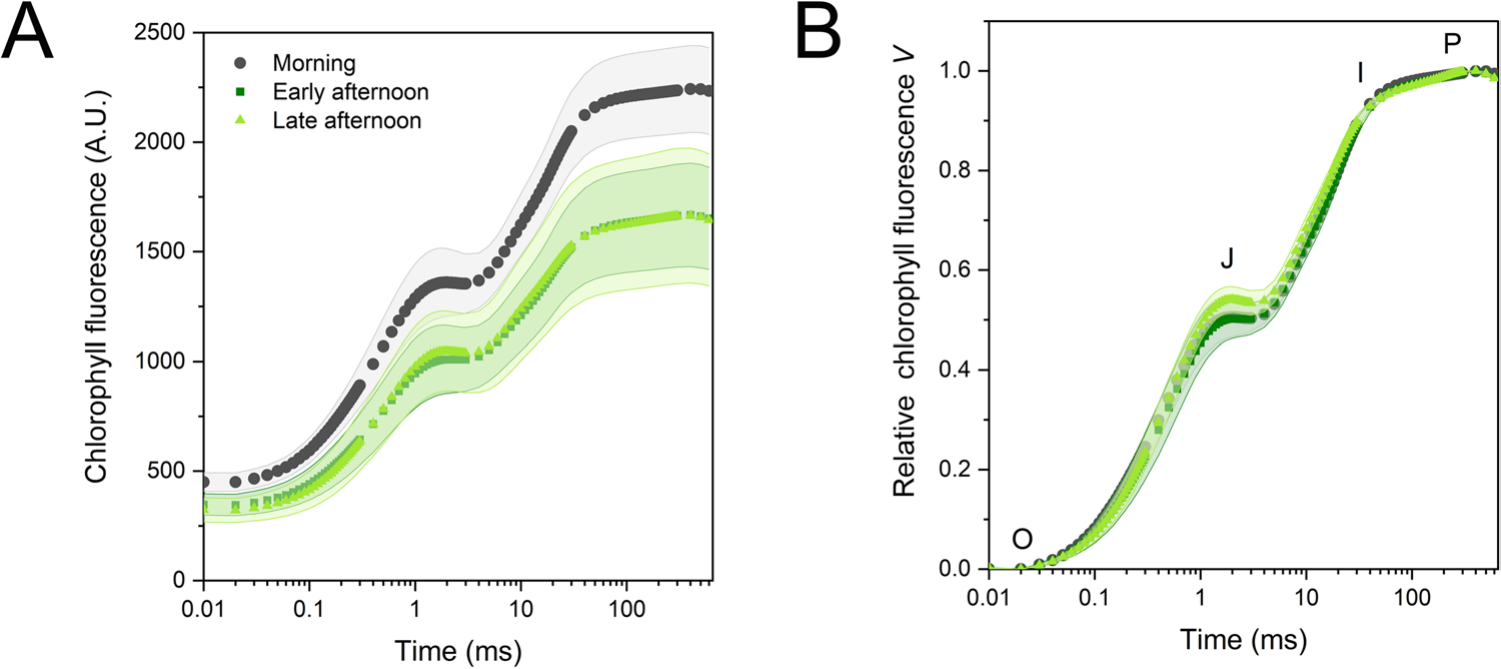
Fast chlorophyll *a* fluorescence transients of *Selaginella martensii* microphylls. In the afternoon, the light green areas were probed. **A** Original average transients. **B** Double normalized transients between the minimum fluorescence at step O and maximum fluorescence at step P. Fluorescence at step O was sampled at 20 µs, while P was reached at ca. 200 ms. The intermediate steps J and I are also indicated at 2 and 30 ms, respectively. Values are means of 24–27 independent measurements. The shaded bands represent one standard deviation

Fast chlorophyll *a* fluorescence is a near-instantaneous reliable method to probe the PSII photochemistry in relation to the functioning of the electron transport chain (Stirbet and Govindjee 2011). In the dark-acclimated state, the electron flow is probed until the ferredoxin-NADP^+^ oxidoreductase (FNR), which is inactivated in the dark and thus cannot allow photosynthetic electrons to reduce NADP^+^ to NADPH and feed the NADPH-consuming metabolism (Schansker et al. 2006). In the induction curves (OJIP transient), the fluorescence signal intensity was lower in the afternoon than in the morning (Fig. 8A). Both *F*_*0*_ and *F*_*M*_ values were accordingly lower (Table 2). The parameters quantifying the PSII photochemistry, *F*_*V*_*/F*_*0*_ and *F*_*V*_*/F*_*M*_, underwent a small decrease in the early afternoon, i.e., the microphylls after the daily peak in irradiance experienced some PSII photoinhibition, which subsequently recovered (Table 2). A more detailed analysis of PSII photochemistry was helped by double normalizing the OJIP transients between O and P steps. The main difference was the relative gain in emission at the J step in the late afternoon samples, leading to a small (− 7.5%), though significant, decrease in ∆*V*_J_, and therefore a lower availability of oxidized plastoquinone (Tóth et al. 2007; Fig. 8B; Table 2). In all samples, the relative amplitude of the I step was very small, as expected for a shade-adapted plant with low photosynthetic capacity and consequently requiring only limited pools of downstream electron carriers (Fig. 8B). No change affected the I step (∆*V*_I_) or the derived parameter ∆*V*_I_/∆*V*_J_. Interestingly, while the estimated electron carriers per electron transport chain (*Sm*) did not change, the turnover number *N*—the times Q_A_ becomes reduced and re-oxidized again until *F*_*M*_ is reached—decreased slightly during the day, confirming the persistence of some reduced plastoquinone even in the dark-acclimated state (Table 2).

**Table 2:** Fluorescence parameters obtained from the OJIP transient analysis of *Selaginella martensii* microphylls in the morning (dark green), early and late afternoon (light green), and after 20-min acclimation to darkness. (mean ± SD; *n* = 24 − 27)

Parameter	Morning	Early afternoon	Late afternoon	ANOVA *P*-value
*F*_*0*_	407 ± 40^a^	318 ± 44^b^	288 ± 50^b^	< 10^−13^
*F*_*M*_	2243 ± 198^a^	1668 ± 236^b^	1665 ± 309^b^	< 10^−13^
*F*_*V*_/*F*_0_	4.02 ± 0.22^ab^	3.88 ± 0.32^b^	4.19 ± 0.26^a^	< 0.001
*F*_*V*_/*F*_*M*_	0.800 ± 0.009^ab^	0.794 ± 0.014^b^	0.807 ± 0.010^a^	< 0.001
∆*V*_*J*_	0.494 ± 0.039^a^	0.503 ± 0.033^a^	0.457 ± 0.026^b^	< 10^−4^
∆*V*_*I*_	0.108 ± 0.012	0.110 ± 0.016	0.102 ± 0.009	0.13
∆*V*_*I*_/∆*V*_*J*_	0.219 ± 0.029	0.222 ± 0.027	0.224 ± 0.021	0.76
*Sm*	14.1 ± 1.6	14.2 ± 1.7	14.1 ± 1.2	0.94
*N*	27.2 ± 2.6^a^	25.5 ± 2.7^ab^	24.1 ± 2.8^b^	< 0.001
*V*_*K*_*/V*_*J*_	0.59 ± 0.04^a^	0.55 ± 0.07^b^	0.43 ± 0.05^c^	< 10^−16^
PSII excitonic connectivity parameters				
*C*	0.207 ± 0.221^a^	0.216 ± 0.186^a^	0.402 ± 0.157^b^	< 0.001
*p*_*2G*_	0.050 ± 0.054^a^	0.054 ± 0.045^a^	0.096 ± 0.037^b^	< 0.01
*p*	0.140 ± 0.179^a^	0.158 ± 0.135^a^	0.278 ± 0.082^b^	< 0.01
*ω*	0.174 ± 0.223^a^	0.198 ± 0.168^a^	0.344 ± 0.101^b^	< 0.01
Specific energy fluxes (per active PSII reaction center)				
ABS/RC	2.93 ± 0.25	2.76 ± 0.44	2.99 ± 0.35	0.051
TR/RC	2.34 ± 0.19^ab^	2.18 ± 0.31^b^	2.41 ± 0.27^a^	< 0.01
ET/RC	1.16 ± 0.12^a^	1.07 ± 0.12^b^	1.10 ± 0.10^ab^	< 0.05
RE/RC	0.251 ± 0.023	0.236 ± 0.029	0.245 ± 0.029	0.14
DI/RC	0.590 ± 0.069	0.577 ± 0.132	0.580 ± 0.088	0.88
Phenomenological energy fluxes (per excited cross section CSo)				
RC/CSo	139 ± 18^a^	117 ± 18^b^	97 ± 15^b^	< 10^−11^
TR/CSo	322 ± 27^a^	248 ± 30^b^	233 ± 40^b^	< 10^−14^
ET/CSo	159 ± 18^a^	125 ± 16^b^	107 ± 21^c^	< 10^−13^
RE/CSo	34.6 ± 4.8^a^	27.5 ± 5.9^b^	24.1 ± 4.3^b^	< 10^−8^
DI/CSo	80.5 ± 8.7^a^	64.4 ± 9.0^b^	55.8 ± 9.3^c^	< 10^−13^

**Fig. 9:**
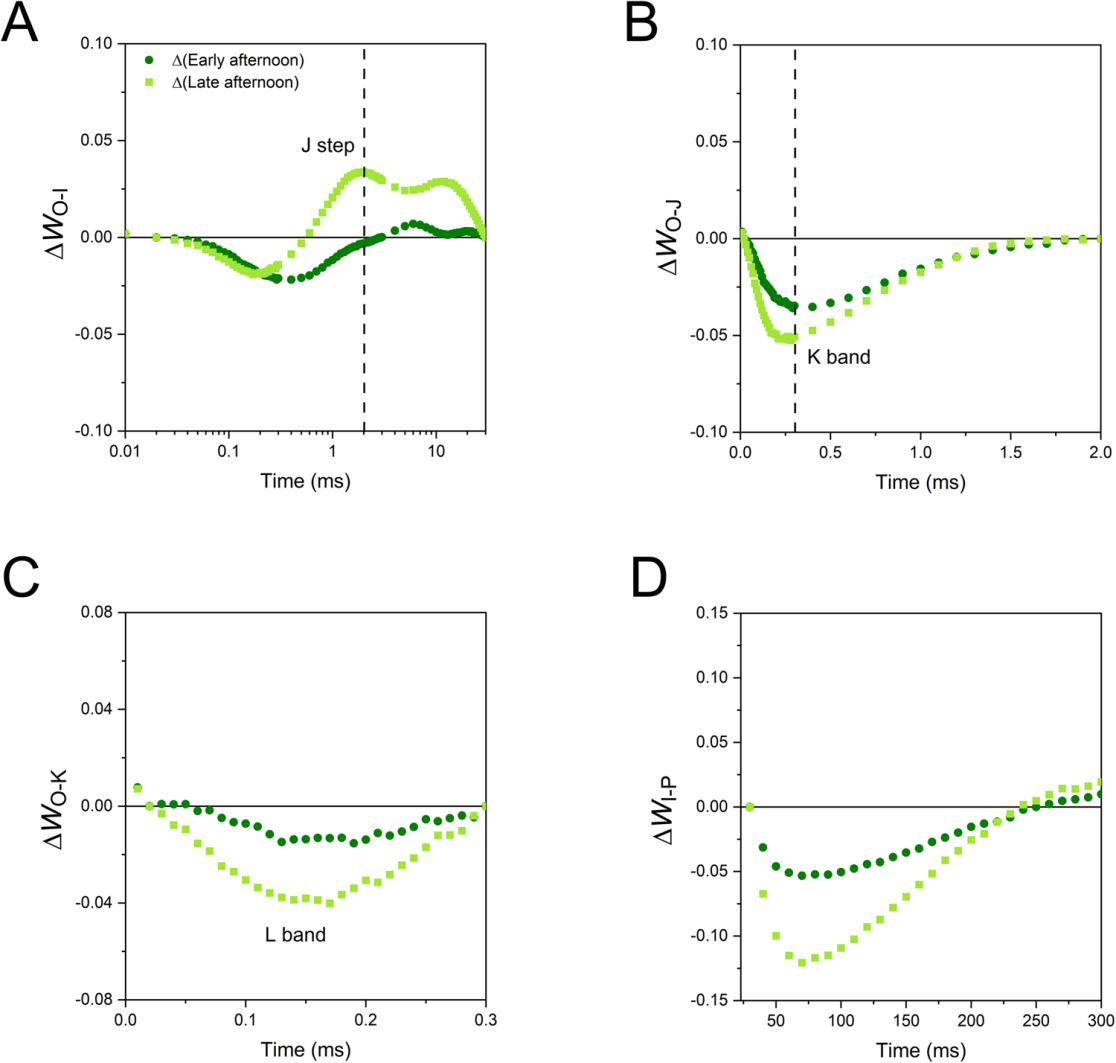
Kinetic differences in chlorophyll fluorescence induction calculated in *Selaginella martensii* microphylls (afternoon minus morning). **A** Kinetic differences between normalized O and I steps, Δ*W*_O-I_. The position of the J step is indicated by the dashed line. **B** Kinetic differences between normalized O and J steps, Δ*W*_O-J_. The state of the photosystem II donor side is probed through the relative intensity of the K band at ca. 300 µs as indicated by the dashed line. **C** Kinetic difference between normalized O and K steps, Δ*W*_O-K_. The region of the L-band highlights changes in the curve sigmoidicity, related to the PSII excitonic connectivity. **D** Kinetic differences between normalized I and P steps, Δ*W*_I-P_. The kinetic differences were calculated from the average transients reported in Fig. 8

To investigate more subtle differences among samples, double normalized kinetics *W* between remarkable steps and difference kinetics ∆*W* were calculated. Analysis of the O-I trait well evidenced the amplitude variation in the J step, but also negative variations of the O-J kinetics (Fig. 9A). This change could be due to variations in PSII antenna organization and/or alterations of the PSII electron donor side, i.e., the oxygen evolving complex. The difference kinetics in the O-J phase revealed a very specific decrease in the relative fluorescence amplitude at ca. 300 µs from O step, which is known as the K band (Fig. 9B; Srivastava et al. 1997). The 27% decrease in the *V*_*K*_*/V*_*J*_ ratio from the morning to the late afternoon indicated that the chloroplasts experienced a progressive recovery of the PSII centers previously damaged at their donor side (Table 1; Srivastava et al. 1997; Brestič et al. 2012; Guo et al. 2020). Analysis of the O-K phase highlighted a specific loss of fluorescence in the late afternoon, corresponding to a more pronounced sigmoidal character of the fluorescence rise (Fig. 9C). According to a common interpretation, the changes in this so-called L band indicate variations in the excitonic connectivity of PSII reaction centers. PSII connectivity can be defined as the transfer of excitation energy from a closed PSII to an open PSII and is mediated by the antenna system shared by neighboring PSII units (Stirbet 2013). A model can be applied that quantifies the curvature of the O-J phase and obtains probabilistic parameters quantifying the PSII connectivity (Strasser and Stirbet 2001). In the morning and early afternoon samples, the overall connectivity probability *p*_*2G*_ was as low as 5% in *S. martensii*; interestingly, the connectivity parameters were nearly doubled in the late afternoon (Table 2). Finally, we analyzed the I-P phase, showing a gradually slowed reduction kinetics of the PSI end acceptor pool in the afternoon samples (Fig. 9D).

The noticeable fluorescence values of the OJIP transient were used to calculate the specific energy fluxes per active PSII reaction center (Strasser et al. 2004; Stirbet and Govindjee 2011). The original model by Strasser et al. (2004) assumes that the PSII units are independent (unconnected), while we found that PSII connectivity strongly increased in the late afternoon. Therefore, the curvature constant *C* was used to correct the specific energy fluxes (Force et al. 2003). Variations were non-significant (*ABS/RC*, *RE/RC*, *DI/RC*), or statistically significant, but biologically minor (*TR/RC*, *ET/RC*).

The lower *F*_*0*_ in the afternoon was the consequence of the diurnal change in the optical properties of a microphyll, in fact *F*_*0*_ can be assumed to approximate the absorbed photon flux per excited cross section (Stirbet and Govindjee 2011). An estimate of the number of reaction centers excited per microphyll section (*RC/CSo*) showed a decrease by 15% and 30% from the morning to the early and late afternoon, respectively (Table 2). Each of the energy fluxes per excited cross section repeated the same difference.

**Fig. 10:**
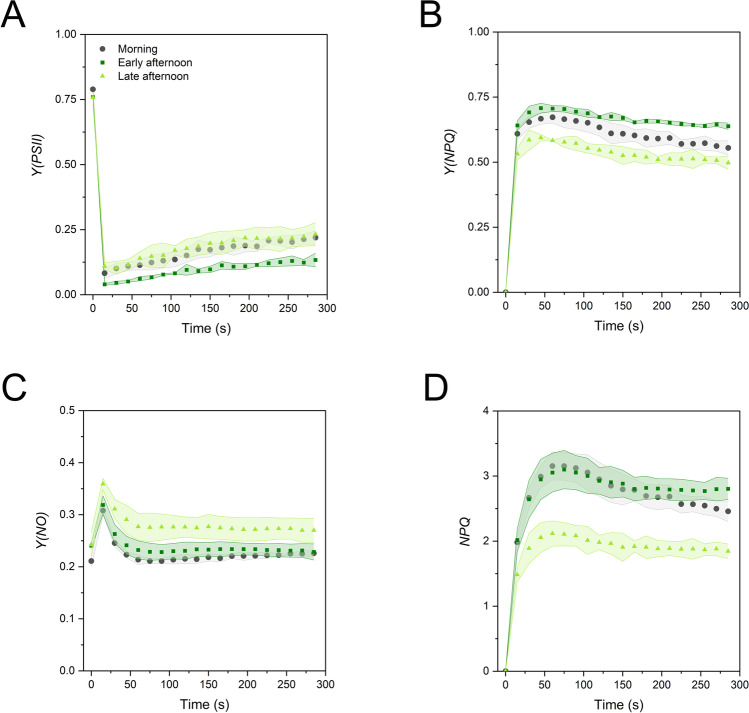
Slow chlorophyll fluorescence induction of *Selaginella martensii* microphylls during the day. Dark-acclimated leaves were probed with 820 μmol photons m^−2^ s^−1^. **A** Actual quantum yield of PSII, *Y(PSII)*. **B** Quantum yield of the regulatory thermal dissipation *Y(NPQ)*. **C** Quantum yield of the non-regulatory energy dissipation, *Y(NO)*. **D** Non-photochemical quenching *NPQ*. Values are means of *N* = 3–4 independent experiments with standard deviations represented as colored area

To evaluate if the variations emerging from the OJIP transients had an impact on the thylakoid functioning in the light, we probed the samples with high light and recorded the slow fluorescence kinetics with a PAM fluorometer. Upon exposure of dark-acclimated samples to high irradiance, *Y(PSII)* underwent an abrupt decrease, followed by the recovery allowed by the progressive activation of the Calvin–Benson–Bassham cycle (Fig. 10A; Tikhonov 2015; Ferroni et al. 2021a). The early afternoon samples were not able to keep *Y(PSII)* at values as high as in the morning and late afternoon microphylls. Their lower *Y(PSII)* was accompanied by a slightly increased induction of the photoprotective regulatory thermal dissipation, as shown by *Y(NPQ)*. In the same samples, the unchanged *Y(NO)* compared to the green microphylls of the morning indicated the successful prevention of the over-reduction of the electron transport chain, in particular of the plastoquinone pool (Fig. 10B-C; Tikkanen et al. 2017). The light green microphylls of the late afternoon showed instead the lowest induction of *Y(NPQ)* and were less capable to control the redox state of the transport chain, leading to consistently higher *Y(NO)* (Fig. 10B–C). Consequently, a most characteristic feature of the late afternoon microphylls was the lower induction of *NPQ* (Fig. 10D), this parameter equating the *Y(NPQ)/Y(NO)* ratio (Ferroni et al. 2014).

## Discussion

The overall micromorphology of the upper epidermal cells in *Selaginella* species, including the special chloroplast shape aligned to the cell bottom, the positioning of the vacuole, the lens-shaped upper tangential cell wall, sometimes associated with silica bodies on its top, can be interpreted as an outstanding outcome of microphyll evolution in extremely shaded rainforest understorey (Sheue et al. 2007; Liu et al. 2020; Shih et al. 2022). In the bizonoplast, the upper parallel arrays of long thylakoid lamellae are one out of many features that promote multiple light reflections within the conical cell, increasing the chances of light absorption in the lower zone of the chloroplast, populated by an extensive granal system (Liu et al. 2020). However, during the afternoon, the chloroplast relocation, the cell shape remodeling, the lateral side-by-side positioning of chloroplast and vacuole, the disappearance of the upper lamellae, the compacting and the “vertical distribution” of the grana system, all impact on the intracellular light paths to limit light absorption.

Signs of slight PSII photoinhibition are envisaged in the morning (donor-side damage, *V*_*K*_*/V*_*J*_) and become clearer in the early afternoon (lower PSII photochemical indexes *F*_*V*_*/F*_*0*_ and *F*_*V*_*/F*_*M*_; Table 2). In the flattened relocated chloroplast, the reduced excitation cross section reduces the probability of further PSII photoinhibition. PSII photoinhibition can be the result of the inactivation of the reaction center (D1 protein) or the oxygen evolving complex (OEC), or both (Kono et al. 2022 and reference therein). Kono et al. (2022) suggested that the OEC damage caused by short light flecks can be better managed by shade plants because its repair is metabolically inexpensive and can occur under very weak light, e.g., at sunset. The acceptor-side damaged PSII is repaired less promptly than the OEC and requires that PSII moves from the protein-crowded appressed domains to the stroma-exposed domains to grant access of PSII to the repair machinery (for review, Li et al. 2018; Kirchhoff 2019). In the bizonoplast, the upper lamellar region is the most directly exposed to light and, during the daily peak in irradiance, it is very logical that it hosts a major part of photoinhibited PSII. Nonetheless, the extensive thylakoid appression, making the upper lamellae like extremely long grana (Ghaffar et al. 2018), hinders a fast PSII repair cycle, and the damaged PSII units will tend to accumulate (Anderson and Aro 1994; Baena-González and Aro 2002). Therefore, the long upper lamellae are conceivably unfavorable to PSII repair. We suggest that in the early afternoon, the transition of the lamellar region to a grana–intergrana architecture is a structural change that responds to a higher demand for PSII repair (Fig. 5C). Because the plants were not exposed to high light and the organelle relocation response occurred seemingly in a random way (Fig. 1), it is difficult to explain the process mechanistically and this aspect was out of the scope of this work (e.g., the role of blue-light perception; for review, Suetsugu and Wada 2020). However, one hypothesis can be that in *S. martensii*, the organelle relocation and thylakoid rearrangement could be induced when a certain threshold of PSII photoinhibition has been reached.

The process of PSII recovery (lower *V*_*K*_*/V*_*J*_, higher *F*_*V*_*/F*_*0*,_ and *F*_*V*_*/F*_*M*_) is complete in the late afternoon when the lamellar region of the thylakoid system has disappeared. Meanwhile, the giant chloroplast has finally acquired a globular shape, though remaining positioned laterally in the cell. In the late afternoon, the thylakoid system morphology is overall in line with a dark-acclimated state but rather extreme as compared to angiosperms (Anderson et al. 2012; Kirchhoff 2019). The degree of thylakoid appression not only is very high, but the thylakoid membranes tend to adhere at their luminal side, and the intergrana thylakoids are also very packed, though not appressed (Fig. 6). The morning-to-afternoon thylakoid shrinkage is evident from the *SRD* analysis. In the morning, the 16 nm *SRD* is relatively low, but still in line with the values between 16 and 21 nm commonly reported in angiosperms (Dekker and Boekema 2005; Kirchhoff et al. 2011; Ünnep et al. 2014). Electron micrographs explain the *SRD* decrease down to 14.3 nm in the late afternoon as the effect of the thylakoid lumen contraction. More in general, in *S. martensii*, the exceptional compactness of the thylakoid system immersed in a very electrondense stroma, indicates that the chloroplast has lowered its water content as compared to the morning condition. Meaningfully, this variation occurs together with a visible reduction in the vacuole volume. The process cannot be explained by a reduced water availability in the humid greenhouse, the relative humidity being always high; and it is not a fixation artifact because it is specific to the afternoon samples and occurs without signs of plasmolysis. The relatively thin cell walls (ca. 170 nm; Ferroni et al. 2021b) go along with the change in the protoplast volume so that the cell loses its conical shape. Therefore, the whole cell reshaping appears as a physiological regulatory response, impacting on the chloroplast. Gu et al. (2022) have recently proposed that the thylakoid appression in grana offers an ultrastructural means to control photosynthesis by reversible swelling and shrinking of lumina based on osmotic water fluxes, which would depend on the water status of the leaf. Particularly, increased thylakoid system compactness can influence the electron flow. The most obvious consequence of the thylakoid shrinkage is on the diffusion of plastocyanin. Assuming a rough estimate of 3.5 nm for both the membrane bilayer thickness and gap partition thickness (Kirchhoff et al. 2011), a decrease in the lumen thickness from ca. 6 to ca. 4 nm would be hardly compatible with the diffusion of plastocyanin, which is ca. 4–5 nm in diameter (Kirchhoff et al. 2011). The expected hindered diffusion of the mobile electron carriers, i.e., plastocyanin but also plastoquinone and ferredoxin (Kirchhoff et al. 2000, 2011; Höhner et al. 2020), is supported in *S. martensii* by the accumulation of reduced plastoquinone in the light-acclimated state (high Y(NO); Tikkanen et al. 2017; Živčak et al. 2019), the incomplete reoxidation of plastoquinone in darkness (low ∆*V*_*J*_; Tóth et al. 2007), and the slowed flow of electrons to ferredoxin (slower I-P rise; Živčak et al. 2015; Ferroni et al. 2022). Another interesting consequence of the rearranged thylakoid system regards the PSII excitonic connectivity, which can be defined as the energetic coupling of neighboring PSII units (see for review Stirbet 2013). In the morning bizonoplast, the PSII units are almost completely isolated from each other: the very low connectivity can depend on an excessive distance between PSII units separated by a lake of LHCII antennae (for the relative abundance of LHCII in *S. martensii*, see Ferroni et al. 2016 and Colpo et al. 2022). In angiosperms (barley) acclimated to the shade, a low PSII connectivity was proposed to have a positive role in PSII photoprotection (Živčak et al. 2014). In *S. martensii*, higher PSII connectivity in the afternoon can be a logical consequence of increased macromolecular crowding in the membrane, which may shorten the distance between neighboring PSII units. Although the functional significance of this modulation remains unknown, we notice that in *S. martensii*, the concurrent modulation of several factors led to a strikingly constant size of the PSII functional antenna (ABS/RC) during the day.

The relocated flattened chloroplast is overall well photoprotected (high NPQ, good electron flow, low PSII connectivity), and its thylakoid rearrangement can very likely support the PSII repair. Differently, probing of the globular chloroplast shows that the organelle is “functionally lazy” from a photosynthetic viewpoint, potentially prone to PSII damage, and moreover, it is structurally unfavorable to PSII repair. However, in the late afternoon, the irradiance is close to zero and the risk of PSII photodamage is inexistent. Some ultrastructural details are instead strongly suggestive of an active remodelling of the thylakoid system through the importation of new materials from the envelope-derived vesicles (Lindquist and Aronsson 2018) and the renewal of thylakoid components allowed by the interaction between membrane and plastoglobules (Rottet et al. 2015; Kirchhoff 2019). The formation of intraplastid peripheral vesicles is an interesting phenomenon in chloroplasts of land plants, initially documented under stress (Morré et al. 1991; Westphal et al. 2003; Karim et al. 2014). Likewise, small vesicles were previously observed in *S. erythropus* kept in darkness for 24 h (Ghaffar et al. 2018). However, a comprehensive analysis across angiosperm species and plastid types by Lindquist et al. (2016) clearly indicates that the formation of ~ 50-nm vesicles is not incidental but a persistent ongoing process in plastids, which is very likely involved in building and maintaining the inner membrane system (Lindquist et al. 2016). For the example of *S. martensii*, we suggest that the envelope-derived vesicles have the same role in the early divergent vascular plant lineage of lycophytes as in angiosperms; particularly, the vesicles can support metabolite import after the sunset to recover the functionality of the thylakoid membranes. The overall organization of the globular chloroplast is that of an organelle in the process of fully re-establishing its function in order to be ready for the subsequent morning.

In conclusion, the giant chloroplast of *S. martensii* epidermal cells is a polymorphic organelle, including unique daily changes in thylakoid architecture. As previously reported, the cup-shaped bizonoplast emphasizes the light-harvesting capacity to sustain carbon assimilation in a low-light, far-red enriched environment. However, in the afternoon, the organelle can acquire a flattened and, subsequently, globular shape, which is very likely more favorable to the renewal of the thylakoid system. The chloroplast repositioning appears as a part of a general reversible reshaping of the epidermal cell, which is possibly mediated by a local loss of water from the protoplast.

## Data Availability

The datasets generated during the current study are available from the corresponding author on reasonable request.
